# The methanolic extract of *Hibiscus sabdariffa *downregulates the relative expression of *Kiss*1 gene in the hypothalamus of Wistar rats: A preliminary report

**DOI:** 10.22038/AJP.2023.22692

**Published:** 2023

**Authors:** Izuchukwu Azuka Okafor, Smart Ikechukwu Mbagwu, Uchenna Somtochukwu Okafor, Johnson Okwudili Nweke, Kingsley Chinemerem Ibeabuchi, Samuel Nduka Ogbonna

**Affiliations:** 1 *Department of Anatomy, Faculty of Basic Medical Sciences, College of Health Sciences, Nnamdi Azikiwe University, Nnewi Campus, PMB 5001, Nnewi, Nigeria*; 2 *Department of Obstetrics and Gynaecology, College of Medicine, University of Ibadan, Ibadan, Nigeria*; 3 *Pan African University of Life and Earth Science Institute (Including Health and Agriculture), PAULESI, University of Ibadan, Ibadan, Nigeria*; 4 *Beth Israel Deaconess Medical Centre, Harvard Medical School, Boston, Massachusetts*; 5 *Hematology Department, Babcock University Teaching Hospital, Ilisan-Remo, Ogun State, Nigeria*; 6 *Diagnostic Laboratory Unit, Medical Centre, Michael Okpara University of Agriculture, Umudike, Abia State, Nigeria*; 7 *College of Nursing Sciences, Our Lady of Lourdes Hospital Complex, Ihiala, Anambra State, Nigeria*

**Keywords:** Antioxidants, Gene expression, Hibiscus sabdariffa, Histology, Hypothalamus, Phytochemistry

## Abstract

**Objective:**

*Kiss*1 gene expression in the rat hypothalamus was investigated following administration of methanolic extract of *Hibiscus sabdariffa* (MEHS) to provide mechanistic evidence for the reproductive effect of the MEHS as a potential regulator of *Kiss*1 gene (which directly controls the hypogonadal axis).

**Materials and Methods:**

This experiment was done using fifteen (15) male rats with average weight of 148 g, randomly grouped into three (3) groups (A-C). Group A was the control group and received no treatment. Group B and C were orally administered with 200 mg/kg and 400 mg/kg of MEHS, respectively. The animals received the extract once a day for twenty-one (21) days. The hypothalamus was harvested on the last day of administration to investigate antioxidant levels, histopathology, and *Kiss*1 gene expression.

**Results:**

The relative expression of *Kiss*1 gene in the group C was downregulated compared to the control group (p=0.023). No significant changes were seen in the antioxidant levels of the groups treated with MEHS when compared to the control. MEHS had no histopathological effects in the hypothalamus at both low (200 mg/kg) and high (400 mg/kg) doses.

**Conclusion:**

High-dose MEHS lowers the expression of the *Kiss*1 gene in the hypothalamus. However, this effect could not be explained by the oxidative profile or histology of the hypothalamus.

## Introduction

The *Kiss*1 gene is a protein coding gene which produces a family of protein products known as kisspeptins, otherwise called metastatin, because of their inhibitory role in cancer progression and metastasis (Gottsch et al., 2009a ▶). Although initially known and described for its role in cancer metastasis, experimental studies over the last decade have revealed the action of the *Kiss*1 gene on other physiological activities of the body, including reproduction (Gottsch et al., 2009a ▶). Kisspeptin, encoded by the *Kiss*1 gene, has been shown to modulate the onset of puberty by enhancing the secretion of the gonadotropic hormones (Luteinizing hormone and Follicle-stimulating hormone) initiated by the release of the gonadotropin-releasing hormone (GnRH) (Ruohonen et al., 2020 ▶). Kisspeptin regulates the activities of the hypothalamus-pituitary-gonadal (HPG) axis by binding to its G-coupled protein receptor called *Kiss*1r (Ruohonen et al., 2020 ▶) and it is predominantly produced in the brain by the hypothalamic nuclei (García-Galiano et al., 2012 ▶). 

*Hibiscus sabdariffa* (HS) is an edible herbaceous plant primarily grown in tropical and sub-tropical areas. Different parts of the plants are commonly used to produce beverages, medicine, and herbal tea (Ali et al., 2005 ▶; Ismail et al., 2008 ▶). The plant contains different active ingredients and nutrients (Okereke et al., 2015 ▶) and it has been demonstrated to possess antioxidant (Obouayeba et al., 2014 ▶), anti-hyperlipidemic (Hajifaraji et al., 2017 ▶), anti-hypertensive (Hopkins et al., 2013 ▶), and diuretic properties (Alarcón-Alonso et al., 2012 ▶; Bakhtiari et al., 2015 ▶). Some active ingredients thought to be responsible for the therapeutic effects of HS include anthocyanins, polyphenols, hibiscus acid (Hopkins et al., 2013 ▶), and flavonoids (Obouayeba et al., 2014 ▶). There exist conflicting reports on the effects of HS extracts or their isolates on the male reproductive system (Orisakwe et al., 2004 ▶; Hanis et al., 2012 ▶; Mahmoud, 2012 ▶; de Arruda et al., 2016 ▶; Nwabufo and Olusanya, 2017 ▶). A study showed HS to improve sperm morphology, sperm concentration, and sperm motility in diabetic animals (Hanis et al., 2012 ▶). However, other studies have reported varying degrees of histological toxicity of the testis, from sperm count reduction (Orisakwe et al., 2004 ▶; de Arruda et al., 2016 ▶), damaging the testicular morphology and ultrastructure (Orisakwe et al., 2004 ▶; Mahmoud, 2012 ▶) to significant alterations of male reproductive hormones - FSH, testosterone (TT), LH, and prolactin (PRL) (Nwabufo and Olusanya, 2017 ▶). 

Disruption or dysregulation of the *Kiss*1/*Kiss*1r system has been shown to disturb normal reproductive activities and linked to the onset of some reproductive diseases (Gorkem et al., 2018 ▶; Silveira et al., 2010 ▶). Testicular alterations are linked to the activities of the reproductive hormones, directly regulated by GnRH which is activated by the *Kiss*1/*Kiss*1r system. A study found a significant negative and positive relationship between kisspeptin levels and serum FSH and TT respectively (Gorkem et al., 2018 ▶). Some phytoextracts have shown varying abilities to alter the expression of the *Kiss*1 gene across several reproductive organs and induce corresponding changes in reproductive activities using animal experiments (Fan et al., 2015 ▶; Okafor et al., 2020a ▶; Okafor et al., 2020b ▶; Feizollahi et al., 2021 ▶). In the search for plants with reproductive potentials, we examined the role of the *Kiss*1 gene expression level in the hypothalamus -the origin of *Kiss*1/*Kiss*1r/HPG axis control-- after the oral administration of methanolic extract of *H. sabdariffa* (MEHS). The hypothalamus has been targeted in this study as the most dominant organ that expresses the *Kiss*1 gene and has direct control on the HPG-axis which plays a huge role in male reproduction (Oakley et al., 2009 ▶). This study hopes to stimulate further investigation into the mechanisms behind the reproductive role of HS, notwithstanding the controversial scientific evidence. 

## Materials and Methods

**Study setting**

This experiment was conducted in the research laboratory of the Department of Anatomy, Nnamdi Azikiwe University. It lasted for about three months**.**

**Plant collection, identification, and extraction**

The dried aerial parts of *H. sabdariffa* were procured from a community market at Nnewi, Anambra state. The botanical identification and authentication were carried out in the Department of Pharmacognosy and Traditional Medicine, Nnamdi Azikiwe University, with the identification number PCG/1474/A/031. The plant calyces were dried under shade and pulverized. One thousand grams of the powdered plant material was used for methanolic extraction as described in a previous study (Okafor et al., 2014 ▶). The extract was freeze-stored at 4⁰C in a refrigerator until use. The extract was made into different doses of solution per milliliter of water on each administration day and administered orally according to the animals' body weight and group treatment doses. 

**Animal procurement, care and handling**

Fifteen (15) male Wistar rats were acquired from the animal house of College of Health Sciences, Nnamdi Azikiwe University and subjected to acclimatization for two (2) weeks (to prevent any intercurrent infection) under standard laboratory housing conditions. The animals were fed without restraint with standard rat chow and water throughout the study duration. Animal health status and welfare were monitored throughout the experiment according to the guidelines of the Federation of European Laboratory Animal Science Associations (FELASA).

**Experimental design**

Fifteen (15) male rats weighing 148 g on average, were divided randomly into three (3) groups (A-C). Group A was the control group and received no treatment. Group B was administered with 200 mg/kg of MEHS orally, and group C got 400 mg/kg of MEHS orally. A solution of 1 g per 20 ml distilled water was constituted with MEHS on each administration day. The concentration needed for every animal was calculated based on the body weight and gotten from the constituted stock. After administration each day, the remaining extract was discarded. Administration of the extract was carried out once every day for 21 days using an oral cannula. The rats' water consumption and feeding patterns before and after the administration and their physical and behavioral changes in response to the administration were monitored. The sample size for this particular study was determined using the resource equation for sample size calculation for animal-based studies (Charan and Kantharia, 2013 ▶), while the doses of the extract administered were selected based on cues from previous studies (Mahmoud, 2012 ▶; Okafor et al., 2020 ▶).

**Animal sacrifice and sample collection**

The animals were subjected to overnight fasting on the last day of extract administration and anesthetized under chloroform. They were sacrificed, and the entire hypothalamus of each animal was harvested and divided into three parts. The division of the hypothalamus was done in median sagittal sections, as shown in another study (Shroder et al., 2020 ▶), to avoid missing out on any hypothalamic nuclei. A portion was fixed in a 10% formal saline for histological processing and analysis, while another part was homogenized and used for oxidative status analysis. The last portion was stored in an RNA protector-containing plain tube before gene analysis. 

**Antioxidant status of hypothalamic tissue**

Glutathione (GSH), Catalase (CAT), and Superoxide Dismutase (SOD) levels were quantified in the hypothalamic tissue harvested to ascertain the antioxidant levels using the tissue homogenate as depicted in an earlier study (Okafor et al., 2018 ▶).

***Kiss*** **1 RNA extraction**

Total RNA was extracted using the Zymo Research (ZR) RNA MiniPrep according to ZR specifications. The extraction protocol used was previously published (Okafor et al., 2020a ▶; Okafor et al., 2020b ▶). A 70 µl volume of the total RNA was extracted, and 10 µl was used for a quality control check on the total RNA extracted, while the rest was transferred into an RNA stable tube supplied by Biomatrica (catalog number 93221-001) for storage at room temperature.

**RNA detection**

The RNA detection was done by agarose gel electrophoresis, that was performed at 90 volts for a period of 30 min with the tetra-source electrophoresis machine (Edvotek, Bethesda, USA), as documented in previous studies (Okafor et al., 2020a ▶; Okafor et al., 2020b ▶). The gel was removed and viewed on the UV transilluminator (Wealtec Dolphin-Doc UV) to capture the genomic bands.

**Reverse transcriptase-polymerase chain reaction (RT-PCR)**

Following the manufacturer's guidelines and specifications, the extracted total RNA was retro-transcribed and amplified using One Taq one-Step RT-PCR kit (catalog number NEB E5315S) by New England BioLabs Incorporation. The MJ research Peltier thermal cycler polymerase chain reaction machine used selected primers to target lymphocyte genes. The RT-PCR protocol was as earlier described (Okafor et al., 2020a ▶; Okafor et al., 2020b ▶). The PCR was started as follows: Reverse transcriptase at 48°C for 30 sec, initial denaturation at 94°C for 1 min, denaturation at 94°C for 15 sec, annealing at Tm°C minus 5 (the lowest melting temperature of each set of *Kiss*1 gene) for 30 sec, extension at 68°C for 1 min, denaturation step for 39 cycles, final extension at 68°C for 5 min and final holding at 4°C, till further use. The' primers' *Kiss*1 gene nucleotide sequence (5'- 3') is - CTACGACTCCTTGTTGCTTTG (forward primer), and TGATCTTCACTGTAGTTGGTGG (reverse primer).

**Electrophoresis of amplified RT-PCR products**

Here, 5 µl of the amplified PCR products and DNA ladder were analyzed on 1% agarose gel containing ethidium bromide in 1X Tris EDTA buffer. The detection protocol used for the amplified RT-PCR product was previously published (Okafor et al., 2020a ▶; Okafor et al., 2020b ▶). Gel electrophoresis was performed for 30 min at 90 volts using the tetra-source electrophoresis machine (Edvotek Bethesda, USA). The *Kiss*1 gene was visualized with the Wealtec Dolphin-Doc UV transilluminator and photographed after electrophoresis. 

***Kiss*** **1 gene relative intensity of expression**

The absolute intensity of expression from the generated gel images across all the experimental groups was calculated with the ImageJ 1.53a software. The software generates the absolute intensity (calculated by the pixel value or percent for each band multiplied by the mean value). The absolute intensity is an integrated measure of the intensity and size of the band. The relative intensity was determined by dividing each sample band's absolute intensity by the standard's absolute intensity.

**Tissue processing**

The hypothalamic tissue samples were sliced down to about 3mm x 3mm for easy study of sections under the microscope and were fixed in 10% formalin. The dehydration of the fixed tissues was done in ascending grades of alcohol 50, 70, 95, and 100% after fixation and cleared in xylene. The samples were stained with hematoxylin and eosin (H& E) and mounted using DPX, after which, the sections were viewed under a light microscope with a magnification of 200x. Photomicrographs of these sections were obtained using the Leica DM 750 digital microscope computer software.

**Statistical analysis**

*Post hoc* LSD test, student's t-test and one-way analysis of variance (ANOVA) were used to test for significant changes across and within groups. The data was analyzed using the IBM statistical package for social science (SPSS) for Windows, version 23 (IBM Corporation, Armonk, New York, USA). Tables and Figures are used to present the data, and values were considered significant at p<0.05.

**Ethical statement**

This study received approval from the Research Ethics Committee of the Anatomy Department, Nnamdi Azikiwe University. The experimental procedures followed in this study complied with Animal Research: Reporting of In Vivo Experiments (ARRIVE) guidelines, National Institutes of Health (NIH) guidelines, and National Health Research ethics committee of Nigeria (NHREC) guidelines for the care and use of laboratory animals. According to the federation of European Laboratory Animal Science Associations (FELASA) guidelines, animal health status was monitored throughout the experiment.  Informed consent was not a requirement for this study.

## Results

**Phytochemical analysis of** ***H. sabdariffa***

The qualitative analysis of HS showed a moderate amount of flavonoids, terpenoid, carbohydrate, reducing sugar, with a trace amount of alkaloid, saponin, and tannin. Steroid and cardiac glycoside were absent (Table 1). The quantitative analysis of some of the major phytochemical constituents of HS showed a relatively high content of flavonoid (7%) and saponin (6.95%) and a relatively small amount of alkaloid (4.2%) and tannin (2.8%) (Table 2).

**Antioxidant levels in the hypothalamus**

**Figure 1:**
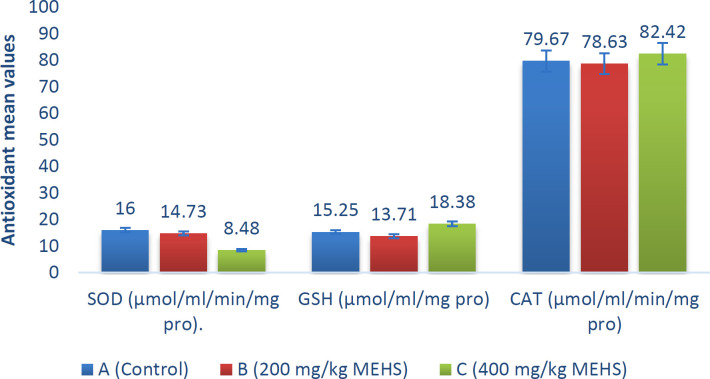
The oxidative status of the hypothalamus of male Wistar rats treated with MEHS. Data were analyzed using one-way ANOVA followed by multiple comparisons using LSD and are expressed as mean±standard deviation (SD). Data were considered significant at (p<0.05). SOD: Superoxide dismutase; GSH: Glutathione; CAT: Catalase. MESH: Methanolic extract of *Hibiscus sabdariffa*

No significant changes were observed in the antioxidant levels in the MEHS-treated groups when compared to the control (Figure 1).

**Effect of MEHS on the relative expression of** ***Kiss*** **1 gene**

A significant downregulation in the relative expression of the *Kiss*1 gene in the hypothalamus of the group C was observed when compared to the control (p=0.023, F=9.545). No significant changes were seen in the group B when compared to the control (Figures 3 and 4).

**Table 1:** Qualitative analysis of *Hibiscus sabdariffa*

Sample	Alkaloid	Saponin	Tannin	Flavonoid	Steroid	Terpenoid	Cardiac glycoside	Protein	Carbohydrate	Reducing sugar
*Hibiscus sabdariffa *	**+**	**+**	**+**	**++**	**--**	**++**	**--**	**+**	**++**	**++**

Key: +Trace/Mildly Present; ++Moderately Present; +++Abundantly Present; -Absent

**Table 2:** Quantitative screening of *Hibiscus sabdariffa*

Alkaloid	Saponin	Flavonoid	Tannin
*2.8%*	6.95%	7%	4.2%

**Figure 2:**
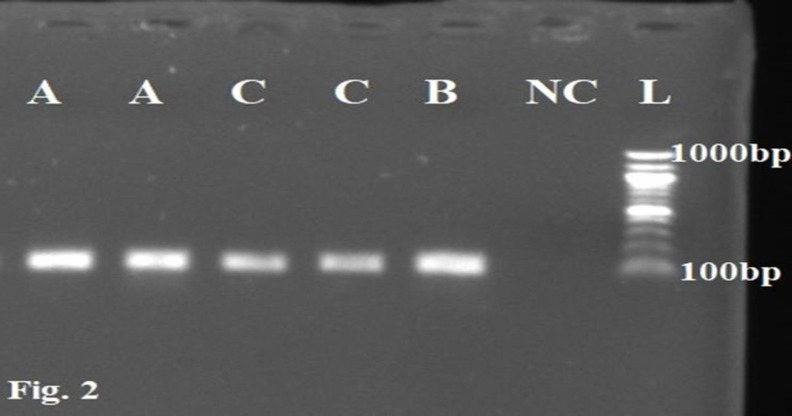
RT-PCR results for *Kiss*1 gene expression on the hypothalamic tissue of male Wistar rats treated with *Hibiscus sabdariffa* extracts analyzed on a 1.0% agarose gel electrophoresis, stained with ethidium bromide. L is a 100bp-1000bp DNA ladder (molecular marker). Samples A, B, and C are positive bands for the expressed *Kiss*1 genes at 100bp representing the study groups. NC is a No template control

**Figure 3:**
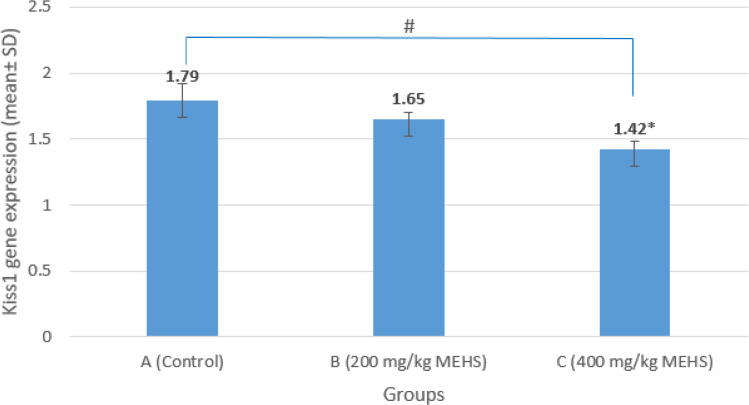
The relative expression of *Kiss*1 gene in the hypothalamus of Wistar rats following administration of MEHS. Data were analyzed using one-way ANOVA followed by multiple comparisons using LSD and are expressed as mean±standard deviation (SD). Data were considered significant at (p<0.05). ^#^p<0.05 means significant compared to the control (group A). MESH: Methanolic extract of *Hibiscus sabdariffa*

**Histopathological findings**

**Figure 4 (plates 1-3):**
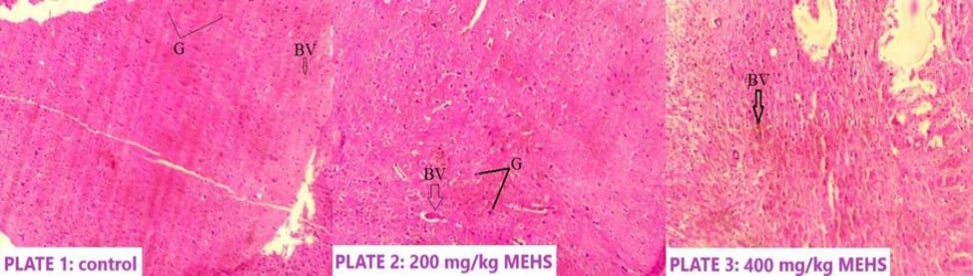
The effect of MEHS on the hypothalamus. Plate 1- Group A, Control; Plate 2-Group B, 200 mg/kg MEHS; Plate 3-Group C, 200 mg/kg MEHS. MEHS: Methanolic extract of *Hibiscus sabdariffa. *H&E, x200. BV: blood vessel; G: glial cells

Figure 4 (plates 1–3) presents the tissue section of the hypothalamus in all the experimental groups. Plate 1 (administered only with distilled water) shows normal hypothalamic tissue. Plate 2 (administered with 200 mg/kg MEHS only) shows a viable hypothalamic tissue with subcortical white matter, blood vessels, and glial cells disposed on a neuropil background. No sign of abnormality was seen**. **Plate 3 (administered with 400 mg/kg MEHS only**)** shows neuronal cell bodies disposed on a neuropil background with no sign of tissue damage. Staining was done using H&E, and photomicrography was taken at a magnification of 200x.

## Discussion

This study showed that MEHS differentially altered the expression of the *Kiss*1 gene in the hypothalamus. On the assessment of the relative expression of the *Kiss*1 gene, it was observed that the administration of 400 mg/kg MEHS caused a significant downregulation of the *Kiss*1 gene in the hypothalamus. In comparison, 200 mg/kg of MEHS did not cause any significant effect (Figure 3). The hypothalamic *Kiss*1 gene is indispensable in developing and maintaining a normal reproductive function (Uenoyama et al., 2016 ▶). One study that evaluated the reproductive and hormonal profiles in *Kiss*1 gene-knockout rats reported an absence of the purge and surge modes of LH and FSH secretion as well as a complete absence of puberty (Uenoyama et al., 2015 ▶). Similar to the present study, a decrease in the hypothalamic *Kiss*1 gene expression has been reported in metabolic imbalance cases that suppress gonadotropin secretion (Wahab et al., 2011 ▶). It could suggest an induced reproductive dysregulation by MEHS. The downregulation caused by MEHS could be proof of a possible regulatory mechanism of HS on male reproduction earlier reported by different studies (Orisakwe et al., 2004 ▶; Hanis et al., 2012 ▶; Mahmoud, 2012 ▶; de Arruda et al., 2016 ▶; Nwabufo and Olusanya 2017 ▶). The current study did not assess the reproductive indices owing to considerable published evidence (Orisakwe et al., 2004 ▶; Mahmoud, 2012 ▶). It is important to note that there was no significant change in the histology of the hypothalamus in the MEHS-treated animals (Figure 4). Thus, MEHS has the potential to affect the *Kiss*1 gene expression without affecting the hypothalamic tissue. Compared to earlier reports of HS-induced histological toxicity in the rat testis, it may be suggested that MEHS effects on the reproductive organs are more severe. Nevertheless, the evidence presented in the current study may not be enough to ascertain this claim. 

The phytochemical analysis of HS shows some important bioactive compounds, including flavonoids (Table 1), which are characterized as phytoestrogen (Moutsatsou, 2007 ▶). Like other endocrine-disrupting compounds (EDCs), phytoestrogen alters the body's endocrine functions by mimicking or even blocking the action of endogenous estrogen (Rogers et al., 2013 ▶). Phytoestrogens exert estrogen-like effects in the body mainly by binding to estrogen receptors (Wocławek-Potocka et al., 2013 ▶). The observed effect of HS in the present study may likely be due to its rich flavonoids content. This is because estrogenic hormones have been shown to inhibit the expression of the *Kiss*1 gene in the arcuate nucleus of the hypothalamus (Gottsch et al., 2009b ▶). This theory appeared to be validated by another study in which soybean isoflavones, a phytoestrogen, was found to not only cause a significant decrease in the expression of hypothalamic *Kiss*1 gene but also induce pubertal delay, as well as decreased serum gonadotropin-releasing hormone and luteinizing hormone (Fan et al., 2015 ▶). 

An assessment of the oxidative profile of the hypothalamus showed no sign of oxidative stress or imbalance, as no significant change (p>0.05) was observed in all the evaluated antioxidative parameters when we compared the MEHS-treated groups to the control (Figure 1). This finding is consistent with our histopathological result which showed no abnormality, tissue damage, or necrosis (Figure 4). 

Overall, this study demonstrated that a high dose of MEHS potentially downregulates the expression of the *Kiss*1 gene in the hypothalamus without affecting the antioxidative profile or histoarchitecture. We suspect that this observed effect of HS could be linked to the presence of active compounds such as flavonoids which have demonstrated estrogen-like properties. The next question that needs to be answered is why the observed MEHS-induced downregulation did not alter the hypothalamus's antioxidant level or histoarchitecture. 

This study presents evidence at the preliminary level, which may be helpful for more complex studies to confirm and consider different aspects of gene expression with real-time qPCR following administration of MEHS. Again, it may be necessary that future studies focus specifically on some hypothalamic area where MEHS effects were more pronounced. This study did not use a house keeping gene but was able to normalize and standardize DNA/RNA concentration and integrity of the samples by loading the exact same amount of nucleic acid material per PCR reaction. However, this may have influenced the outcome and interpretation. 

MEHS-induced down regulation of the *Kiss*1 gene expression in the hypothalamus is not mediated by antioxidant or histopathological mechanisms. MEHS shows preliminary evidence for possible isolation of bioactive phytochemicals beneficial for targeting the *Kiss*1 gene downregulation against some reproductive disorders modulated by the *Kiss*1/*Kiss*1r system. Further studies need to be carried out to fully understand the underlying mechanism involved in the reported activity of MEHS and isolate important active constituents that drive the seen effects.

## Conflicts of interest

The authors have declared that there is no conflict of interest.
